# Is Senescence-Associated β-Galactosidase a Reliable *in vivo* Marker of Cellular Senescence During Embryonic Development?

**DOI:** 10.3389/fcell.2021.623175

**Published:** 2021-01-28

**Authors:** José Antonio de Mera-Rodríguez, Guadalupe Álvarez-Hernán, Yolanda Gañán, Gervasio Martín-Partido, Joaquín Rodríguez-León, Javier Francisco-Morcillo

**Affiliations:** ^1^Área de Biología Celular, Departamento de Anatomía, Biología Celular y Zoología, Facultad de Ciencias, Universidad de Extremadura, Badajoz, Spain; ^2^Área de Anatomía y Embriología Humana, Departamento de Anatomía, Biología Celular y Zoología, Facultad de Medicina, Universidad de Extremadura, Badajoz, Spain

**Keywords:** cell death, cell senescence, retina, development, histochemistry, limb

## Abstract

During vertebrate embryonic development, cellular senescence occurs at multiple locations. To date, it has been accepted that when there has been induction of senescence in an embryonic tissue, β-galactosidase activity is detectable at a pH as high as 6.0, and this has been extensively used as a marker of cellular senescence *in vivo* in both whole-mount and cryosections. Such senescence-associated β-galactosidase (SA-β-GAL) labeling appears enhanced in degenerating regions of the vertebrate embryo that are also affected by programmed cell death. In this sense, there is a strong SA-β-GAL signal which overlaps with the pattern of cell death in the interdigital tissue of the developing limbs, and indeed, many of the labeled cells detected go on to subsequently undergo apoptosis. However, it has been reported that β-GAL activity at pH 6.0 is also enhanced in healthy neurons, and some retinal neurons are strongly labeled with this histochemical technique when they begin to differentiate during early embryonic development. These labeled early post-mitotic neurons also express other senescence markers such as p21. Therefore, the reliability of this histochemical technique in studying senescence in cells such as neurons that undergo prolonged and irreversible cell-cycle arrest is questionable because it is also expressed in healthy post-mitotic cells. The identification of new biomarkers of cellular senescence would, in combination with established markers, increase the specificity and efficiency of detecting cellular senescence in embryonic and healthy mature tissues.

## Cellular Senescence

The study of cellular senescence was initiated by Hayflick and Moorhead (1961). Those authors reported that human fibroblasts isolated from embryonic tissues cease to proliferate after a limited number of cell divisions. We now know that the loss of proliferative activity is the consequence of progressive shortening of telomeres in each replicative round (for a review, see Bernadotte et al., 2016). Currently, we have learnt a lot about the stimuli that trigger cellular senescence and about the intracellular effector pathways that execute this process. Cellular senescence is characterized by a prolonged and irreversible cell-cycle arrest with secretory features, macromolecular damage, and altered metabolism (Campisi et al., 2011; Rodier and Campisi, 2011; Childs et al., 2015; Hernandez-Segura et al., 2018; Gorgoulis et al., 2019). The cells that exhibit these features of senescence normally accumulate in aging tissues, further linking this cellular state with the aging process in general (Dimri et al., 1995). Cellular senescence is now also considered to be a suppressive mechanism against oncogenesis (Lee and Lee, 2019), acting to block proliferation in cells with oncogenic mutations (Campisi et al., 2011). Recent studies also describe beneficial effects of cellular senescence during embryonic development, tissue repair and regeneration, and cellular reprogramming (Czarkwiani and Yun, 2018; Rhinn et al., 2019). Senescence during embryonic development is involved in tissue remodeling and is usually linked to areas of cell death that arise in degenerating structures (Muñoz-Espín and Serrano, 2014). Therefore, senescent cells can be detected from embryogenesis (when they contribute to tissue development) to adulthood (when they prevent the propagation of damaged cells and contribute to tissue repair and tumor suppression).

Senescent cells are metabolically active and possess some features *in vitro* and *in vivo* which are known biomarkers of cellular senescence (Bernadotte et al., 2016; Matjusaitis et al., 2016; Hernandez-Segura et al., 2018; Wang and Dreesen, 2018). One of the most used methods to assess cellular senescence is the detection of β-galactosidase (β-GAL) activity at pH 6.0, a histochemical assay that is called “senescence associated β-GAL” (SA-β-GAL), because it labels senescent cells, both *in vivo* and *in vitro* (Dimri et al., 1995). However, intense β-GAL labelling at pH 6.0 could also reflect an alteration in lysosomal number or activity in non-proliferating cells (Yegorov et al., 1998) or in terminally differentiated cells such as neurons (Piechota et al., 2016, inter alia). It has been demonstrated that the so-called SA-β-GAL increases with aging in subsets of neurons in different brain areas, but this enzymatic activity is also present in specific populations of neurons in very young mice (for a review, see Walton and Andersen, 2019). In this sense, it is known that neurons become post-mitotic very early in development, and a recent study performed in our laboratory clearly demonstrated that β-GAL activity at pH 6.0 is intense in recently differentiated ganglion cells in the developing avian retina (de Mera-Rodríguez et al., 2019).

In the present communication, we discuss the reliability of SA-β-GAL activity in identifying cellular senescence *in vitro* and *in vivo*. We first focus on the description of the cell-type-specific labeling of SA-β-GAL in embryonic structures of different vertebrates. Then, we consider the possible relationship between areas of intense SA-β-GAL-staining and areas affected by massive cell death in different embryonic structures. Finally, we compare the staining pattern of SA-β-GAL activity with the distribution of other markers of cell senescence, cell death, and cell differentiation in the developing visual system of vertebrates.

## SA-β-GAL Histochemistry

Lysosomal β-GAL cleaves β-D-galactose residues in β-D-galactosides. Detectable β-GAL activity is the most extensively used marker for senescent or aging cells whether in culture or in mammalian tissues (Dimri et al., 1995; Debacq-Chainiaux et al., 2009). Specifically, at pH 6.0, the β-GAL enzyme hydrolyses 5-bromo-4-chloro-3-indoyl-β-d-galactopyranoside (X-gal), a colorless, soluble compound consisting of galactose linked to an indole. This reaction releases a deep blue, insoluble product on the cell culture or in the tissue. This histochemical technique is distinct from the acidic β-GAL activity, present in lysosomes of all non-senescent cells and detectable at pH 4.0 (Kuilman et al., 2010). However, it has been demonstrated (Lee et al., 2006) that lysosomal β-GAL is the origin of SA-β-GAL activity, with the increased SA-β-GAL activity detected in senescent cells clearly being a result of the increased expression of *GLB1*, the gene encoding the lysosomal enzyme. Furthermore, increased lysosomal biogenesis has been described in senescent cells (Kurz et al., 2000; Severino et al., 2000; Hernandez-Segura et al., 2018).

Nonetheless, some researchers have clearly shown that in some cases β-GAL activity is not indicative of senescence. For instance, it has been described that β-GAL activity at pH 6.0 might reflect the activity of autophagy due to the intense biogenesis of lysosomes that occurs during this process (Young et al., 2009). Intense β-GAL histochemical signals have also been reported in the visceral endoderm at early stages of mouse embryo development (Huang and Rivera-Pérez, 2014) and in the luminal cells of the duodenum (Going et al., 2002). Furthermore, endogenous β-GAL activity at pH 6.0 is detected cytochemically in immortalized cultured cells and macrophage-like cells (Yegorov et al., 1998), in macrophages and osteoclasts in mature tissues (Bursuker et al., 1982; Kopp et al., 2007; Hall et al., 2017), and in confluent non-transformed fibroblast cultures (Severino et al., 2000). A recent study has shown that Purkinje, choroid plexus, heart muscle, intestinal, and pancreatic cells in mammalian tissues are strongly positive for SA-β-GAL activity (Raffaele et al., 2020). Also, serum starvation and confluent culture increase SA-β-GAL activity (Yang and Hu, 2005). In the case of the nervous system, it has been proposed that β-GAL activity at pH 6.0 might be detected in differentiated neurons (Piechota et al., 2016, see below), and even at early stages of development (de Mera-Rodríguez et al., 2019, see below). All these findings indicate that the reliability of the “so-called” SA-β-GAL assays is questionable because, since the enzyme is not always specific for cell-aging, this histochemical technique is insufficient to characterize cellular senescence.

Senescent cells also show additional signature features that could be used in combination with SA-β-GAL to identify the state of senescence. Morphologically, cultured senescent cells become flat, large, multi-nucleated, and vacuolated (Denoyelle et al., 2006). Recently, it has been shown that senescent cells in tissues of aged mice are larger than non-senescent cells (Biran et al., 2017) and that senescent alveolar progenitors show abnormal elongated morphology (Kobayashi et al., 2020). In contrast, in embryonic living tissues, senescent cells usually have a normal morphology (Muñoz-Espín and Serrano, 2014). Senescence-associated heterochromatic foci (SAHF) contain trimethylation at Lys9 of histone 3 (H3K9me3), heterochromatin protein 1 homologue-γ (HP1 γ), and macroH2A (Sharpless and Sherr, 2015; Bernadotte et al., 2016; Wang and Dreesen, 2018; Rhinn et al., 2019). Senescent cells undergo long-term exit from the cell cycle, lacking markers of cell proliferation such as Ki67 or PCNA (Rhinn et al., 2019). They also activate tumor suppressor networks, including p16^*INK4A*^ and p19^*ARF*^ which function by activating the Rb protein and the p53 transcription factor, respectively (Lowe and Sherr, 2003).

These are among the most commonly used markers of cell senescence in cultured cells and in aged or pathological tissues. However, the analysis of senescence signatures in some populations of cells in the developing embryo shows enhanced expression of the cell cycle inhibitor p21, but not that of typical markers of senescence in aging including p53, p16, or p19 (Muñoz-Espín et al., 2013; Storer et al., 2013).

## SA-β-GAL Staining in the Developing Embryo

### SA-β-GAL Staining in Whole Mount Embryos

**FIGURE 1:**
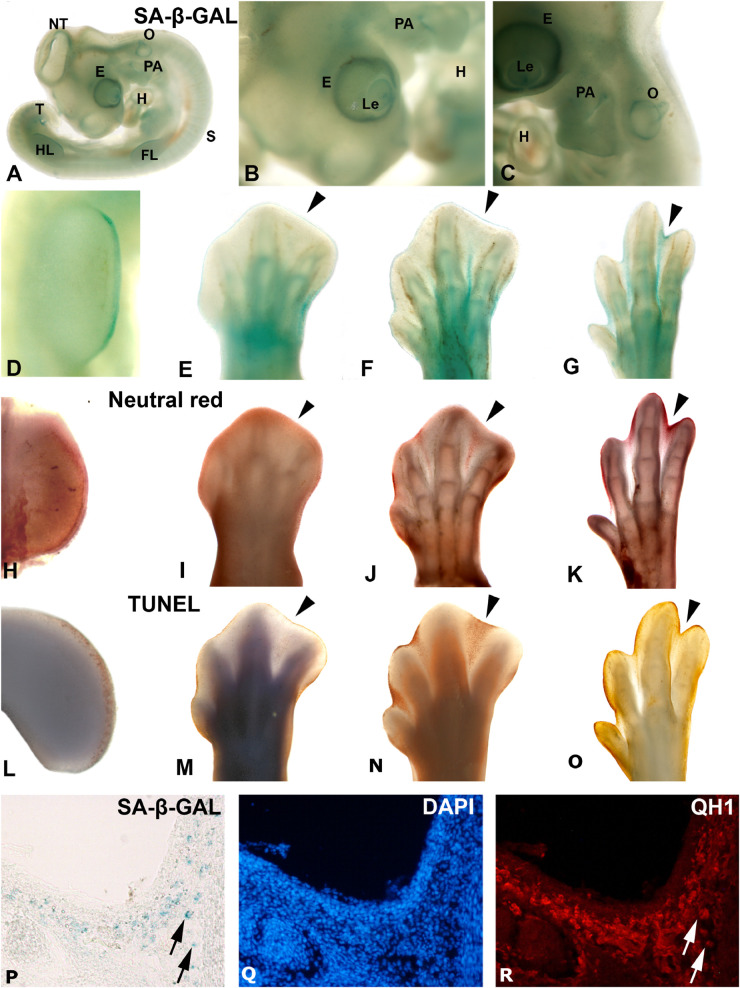
Areas segmenting SA-β-GAL activity and apoptosis during avian embryonic development. Detection of SA-β-GAL activity in embryonic day 3.5 **(A,B)** and E4 **(C)** chicken embryos, and E3.5 **(D)**, E6 **(E)**, E7 **(F)**, and E8 **(G)** hindlimbs. Neutral red staining for cell death detection in E3.5 **(H)**, E6 **(I)**, E7 **(J)**, and E8 **(K)** hindlimbs. TUNEL assay for apoptosis detection in E4 **(L)**, E6 **(M)**, E7 **(N)**, and E8 **(O)** hindlimbs. Labeling of AER can be noted in **(D**,**H**, and **L)**. Arrowheads in **(E–G)**, **(I–K)**, and **(M–O)** point to the third interdigital space during the establishment of cell senescence and the progression of interdigital programmed cell death. SA-β-GAL histochemistry **(P)** and QH1 immunostaining **(R)** label macrophages in the interdigital mesenchyme of quail at stage 36. DAPI staining **(Q)** shows the structure of the interdigital space. E, eye; Fl, forelimb; H, heart, HL, hindlimb, Le, lens; NT, neural tube, O, otic vesicle; PA, pharyngeal arches; T, tail bud.

Programmed cellular senescence is an essential process during vertebrate embryonic development (for reviews, see Muñoz-Espín and Serrano, 2014; Czarkwiani and Yun, 2018; Rhinn et al., 2019; Da Silva-Álvarez et al., 2019; Sacco et al., 2021). SA-β-GAL labeled cells are found stereotypically in well-defined time windows during embryonic development (Muñoz-Espín et al., 2013; Storer et al., 2013). SA-β-GAL staining in whole-mount mammalian embryos clearly shows that abundant labeled cells accumulates in the developing limbs, nails, focal areas of the skin, the tip of the tail, heart, eye tissues, inner ear, olfactory epithelium, and the closing neural tube (Muñoz-Espín et al., 2013; Storer et al., 2013; Zhao et al., 2018), with a staining pattern similar to that observed in whole-mount chicken embryos (Figures 1A–C) (cf. Storer et al., 2013; Lorda-Díez et al., 2015, 2019; Gibaja et al., 2019). More recently (Da Silva-Álvarez et al., 2020), putative senescent cells during zebrafish development have been characterized in detail by using SA-β-GAL staining in whole mounts, with the strong activity being detected in the yolk, cloaca, central nervous system (CNS), intestine, liver, pronephric ducts, and lens.

### SA-β-GAL Staining in Degenerating Embryo Areas: The Case of the Developing Limb

The distribution of SA-β-GAL positive areas in the whole embryo strongly correlates with the distribution of apoptotic cells. Thus, intense SA-β-GAL staining is detected in degenerating structures such as the pronephros of fish (Villiard et al., 2017) and amphibians (Davaapil et al., 2017; Villiard et al., 2017), and in the mesonephros of birds (Nacher et al., 2006) and mammals (Muñoz-Espín et al., 2013; Da Silva-Álvarez et al., 2018). The pattern of cell death in the developing avian heart also strongly correlates with SA-β-GAL staining (Lorda-Díez et al., 2019), as do areas of cell death in the somites, tail bud, and CNS (Muñoz-Espín et al., 2013; Storer et al., 2013). In the case of the developing otic vesicle in the chicken, SA-β-GAL labeled cells are associated with areas of increased apoptosis (Figures 1A–C) (Gibaja et al., 2019; Magariños et al., 2020). In mice, it has been found that senescence in the endolymphatic sac has a morphogenetic role analogous to that of apoptosis (Muñoz-Espín et al., 2013).

The chronotopographical coincidence of cell senescence and apoptosis in the embryo is even more evident in the developing limb. Since the scope of this article is discussion of the reliability of β-galactosidase staining as an *in vivo* marker for senescence detection, we would encourage the reader to refer to the detailed review of limb apoptosis and senescence in another article included in this issue authored by Montero et al. (2020). At early stages of limb development, limb outgrowth is controlled by an epithelial thickening at the distal tip of the structure – the apical ectodermal ridge (AER) (Figures 1D,H,L). The cells of this signaling center proliferate together with the other tissues in the limb bud but undergo apoptosis from soon after the bud’s formation until its disappearance when all the phalanxes have been formed (Rodríguez-León et al., 2013). Concomitant with the apoptotic process (Figures 1H,L), AER cells also exhibit SA-β-GAL activity (Figure 1D) and express different markers of cellular senescence such as p21 (Storer et al., 2013; Muñoz-Espín et al., 2013; Lorda-Díez et al., 2015; Li et al., 2018). In mice, a lack of p21 activity results in senescence defects and AER structural deficiencies, thereby impairing normal limb development (Storer et al., 2013). Indeed, during limb development in the mouse, strong SA-β-GAL activity is detected in the AER at early stages but almost disappears at more advanced stages (Li et al., 2018). These last authors suggest that some senescent cells in this region of the developing mouse limb undergo apoptosis and are removed by phagocytosis. However, a sub-population of these embryonic cells positive for ß-GAL activity and p21 expression remains in the limb tissues after birth, and re-enter the cell cycle, proliferating *in situ* (Li et al., 2018). These results could suggest that some cells in the population are non-senescent cells or a degree of plasticity in the process of cellular senescence during development. Future works are needed to clarify if cellular senescence during development is a process that have some unique features that distinguish it from the adult cellular senescence.

But this is not the only structure that undergoes apoptosis during limb development. The anterior and posterior margins of the limb bud, as well as the interdigital areas, enter the apoptosis program to sculpt the final shape of the organ (reviewed by Montero et al., 2020). Moreover, the interdigital regression during digit formation has been regarded as an excellent model for the study of how cellular senescence and programmed cell death are related (Lorda-Díez et al., 2015; Sanchez-Fernandez et al., 2019). Apoptotic interdigital areas can be observed for approximately 48 h in the developing chicken limb – from 6.5 to 8.5 days of incubation (Montero and Hurlé, 2010; Lorda-Díez et al., 2015). The classical way to detect the interdigital apoptotic pattern is to use vital dyes such as Neutral Red (Figures 1H–K). Staining with this dye allows visualization of tissue removal in the AER (Figure 1H) and the progression of interdigital cell death (Figures 1K,L). But it cannot distinguish apoptosis (the active and programmed process of death in the cells of a developing tissue) from necrosis (the passive death of cells due, for instance, to toxicity or cellular damage). For this reason, since the decade of the 1990s, the TUNEL assay has been used to ensure the detection of apoptotic areas during limb development (Figures 1L–O). The population of apoptotic cells detected by the two techniques in the interdigital areas coincides with the pattern of SA-β-GAL labeling in these regions (Figures 1E–G), evidence for the correlation of the two processes during limb development. Indeed, different studies have shown that cellular senescence and apoptosis coincide during limb development and are essential for proper interdigital regression (Muñoz-Espín et al., 2013; Lorda-Díez et al., 2015; Montero et al., 2016).

Besides the activation of SA-β-GAL activity, another essential feature of cell senescence is cell cycle arrest. In the case of limb interdigital regression, this is connected to up-regulation of genes such as p21, p63, and p73 which block cell cycle progression (Lorda-Díez et al., 2015; Sanchez-Fernandez et al., 2020). Also, various members of the Btg/Tob tumor suppressor gene family are expressed in the interdigital space during the progression of programmed cell death, and overexpression of Btg2 in the early limb mesenchyme results in an anti-proliferative and pro-apoptotic effect on the tissue, leading to the formation of shortened limbs (Lorda-Díez et al., 2015). Interestingly, the expression of these tumor suppressor genes in chicken and mouse embryos, species with free digits, is up-regulated during the course of programmed cell death in the interdigital spaces, but is down-regulated, or maintained at stable levels, in the interdigital areas of the duck which maintains webbed digits in adulthood (Lorda-Díez et al., 2015).

Another important feature of cell senescence is the up-regulation of different components of the senescence-associated secretory phenotype (SASP), namely, different matrix metalloproteinases, IgfBP5, TNF signaling pathway members, and interleukin 8 (Rhinn et al., 2019). Several of these SASP members are up-regulated in the interdigital areas (Lorda-Díez et al., 2015) and in the AER (Storer et al., 2013) when cells are dying by apoptosis.

All this evidence, namely detection of SA-ß-GAL activity and upregulation of cell cycle inhibitors as well as SASP components, supports that SA-β-GAL staining is detected in senescent cells during development in apoptotic areas like those detected in the tetrapod limb. Nevertheless, other populations of non-senescent cells with a high lysosomal mass and/or increased β-GAL lysosomal activity, such as QH1-immunoreactive macrophages in the quail interdigital area, also show strong SA-β-GAL labeling (Figures 1P–R), coinciding with previous studies in mice (Hall et al., 2017).

### SA-β-GAL Staining in the Nervous System: The Case of the Developing and Mature Retina

The analysis of the SA-β-GAL labeling in the developing and mature CNS is a topic of controversy in the field of neuronal senescence. SA-β-GAL activity has been used to detect putative senescent cells in the aging brain of mice (Ori et al., 2015), in cultures of primary cortical neurons (Chernova et al., 2006), and in cerebellar granule neurons (Bhanu et al., 2010). SA-β-GAL is detected cytochemically in neurons of the hippocampus, and its activity increases in old animals (Geng et al., 2010; Piechota et al., 2016) and in prolonged-cultured hippocampal neurons (Dong et al., 2011; Xu et al., 2019). Furthermore, this enzymatic activity is greater in hippocampal neurons after an injury (Tominaga et al., 2019) and in the Purkinje cells of adult mice (Jurk et al., 2012). Neurodegenerative diseases also cause increased SA-β-GAL staining in astrocytes, oligodendrocytes, and microglial cells (Kritsilis et al., 2018). In sum therefore, SA-β-GAL activity increases in mammalian neurons and glial cells during the aging process and under pathological conditions.

**FIGURE 2:**
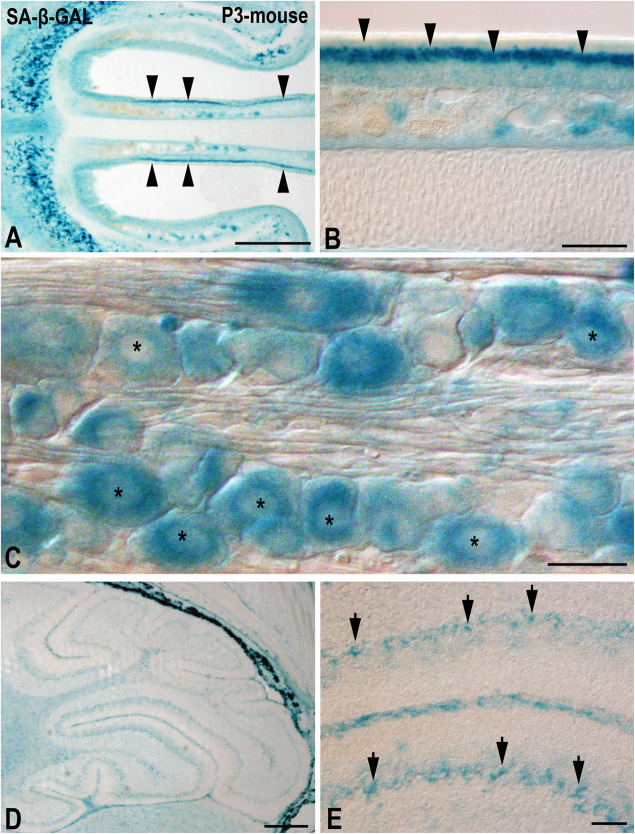
The presence of SA-β-GAL activity in the postnatal day P3 mouse head tissue. Horizontal **(A,B)** and sagittal **(C–E)** cryosections were treated with SA-β-GAL histochemistry. **(A,B)** Intense SA-β-GAL signal is found in the intermediate layer of the olfactory epithelium (arrowheads). **(C)** Strong SA-β-GAL staining is detected in sensory neurons in the trigeminal ganglion (asterisks). **(D,E)** SA-β-GAL activity is detected in the cerebellum, mainly in the Purkinje cell layer (arrows). Scale bars: 200 μm **(A,D)**, 50 μm **(B,E)**, and 20 μm **(C)**.

Intense SA-β-GAL activity has also been detected in several types of neurons in very young mice (1–3 months old) *in vivo* and *in vitro* (Jurk et al., 2012; Piechota et al., 2016; Bussian et al., 2018; Musi et al., 2018; Raffaele et al., 2020). In this sense, intense SA-β-GAL staining is detected in several populations of neurons in horizontal cryosections of the head of a 3-day-old mouse. Strong SA-β-GAL signal is detected in the intermediate layer of the olfactory epithelium, the region where the olfactory sensory neurons are located (Figures 2A,B), but also in neurons of the trigeminal ganglion (Figure 2C) and in the cerebellar Purkinje cells (Figures 2D,E). Therefore, the detection of this enzymatic activity in the CNS at relatively early stages of the postnatal life suggests that it cannot be attributed solely to cell senescence either *in vivo* or *in vitro*. Given this scenario, it is quite possible that β-GAL activity at pH 6.0 could be detected in neurons even in embryonic tissues.

The vertebrate retina contains six well-known types of neurons – ganglion, amacrine, horizontal, bipolar, cone, and rod cells. Much is known about the intrinsic programs (mainly coded by transcription factors, but also by extrinsic factors such as growth factors) which are involved in retinogenesis and in the maintenance of neuronal phenotypes (Álvarez-Hernán et al., 2013, 2018, 2019, 2020; Xiang, 2013; Bejarano-Escobar et al., 2014, 2015). Furthermore, several phases of cell death have been reported to occur during development of the vertebrate retina (Cook et al., 1998; Knabe et al., 2000; Péquignot et al., 2003; Francisco-Morcillo et al., 2004, 2014; Rodríguez-Gallardo et al., 2005; Chavarría et al., 2007, 2013; Valenciano et al., 2009; Bejarano-Escobar et al., 2011, 2013). Therefore, the embryonic retina constitutes an excellent model with which to study whether SA-β-GAL activity is linked with neuronal differentiation processes and/or with cell death areas.

Previous studies have shown that SA-β-GAL histochemistry assay shows staining of the retinal pigment epithelium in the developing avian retina (de Mera-Rodríguez et al., 2019) and in the mature retina of rats (Lamoke et al., 2015) and primates (Mishima et al., 1999), including humans (Hjelmeland et al., 1999; Matsunaga et al., 1999). In this sense, some retinal pathologies are linked to cellular senescence that occurs in the retinal pigment epithelium (Kozlowski, 2012). Specific SA-β-GAL enzymatic activity is observed in human retinal blood vessels, mainly in the endothelial and smooth muscle cells (López-Luppo et al., 2017), and intense SA-β-GAL activity is observed in the microvasculature of diabetic rats (Lamoke et al., 2015). With regard to SA-β-GAL activity in neural retinal tissue, in the mouse at early postnatal stages, SA-β-GAL staining is enhanced in retinal ganglion cells and subpopulations of neurons dispersed throughout the inner nuclear layer (INL) (Oubaha et al., 2016). It has also been reported that, in the adult mouse, SA-β-GAL activity in retinal ganglion cells might be increased by acute intraocular pressure induced ischæmic injury (Li et al., 2017). Other diseases, such as retinopathy of prematurity and proliferative diabetic retinopathy, also lead to cell senescence of several types of retinal neurons (Sapieha and Mallette, 2018).

**FIGURE 3:**
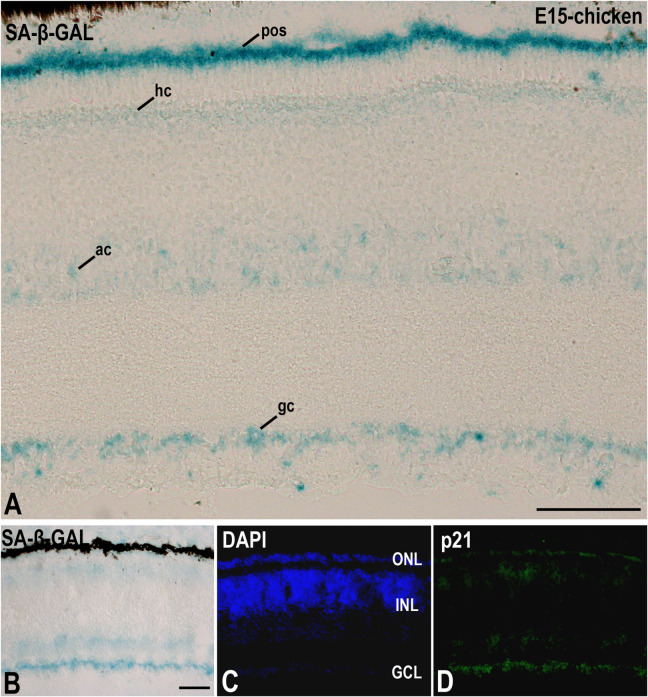
The presence of SA-β-GAL activity in the embryonic day E15 chicken retina. Cryosections of retinas were treated with SA-β-GAL histochemistry **(A,B)** and antibodies against p21 **(B–D)**. DAPI staining shows the laminated structure of the retina **(C)**. SA-β-GAL staining is found in the photoreceptor outer segments and in subpopulations of amacrine and ganglion cells **(A,B)**. The horizontal cell layer appears faintly labeled **(A,B)**. p21 immunostaining strongly correlates with the SA-β-GAL labeling pattern. ac, amacrine cells; gc, ganglion cells; GCL, ganglion cell layer; hc, horizontal cells; INL, inner nuclear layer; ONL, outer nuclear layer; pos, photoreceptor outer segments. Scale bars: 50 μm (**A** and **B–D**).

**FIGURE 4:**
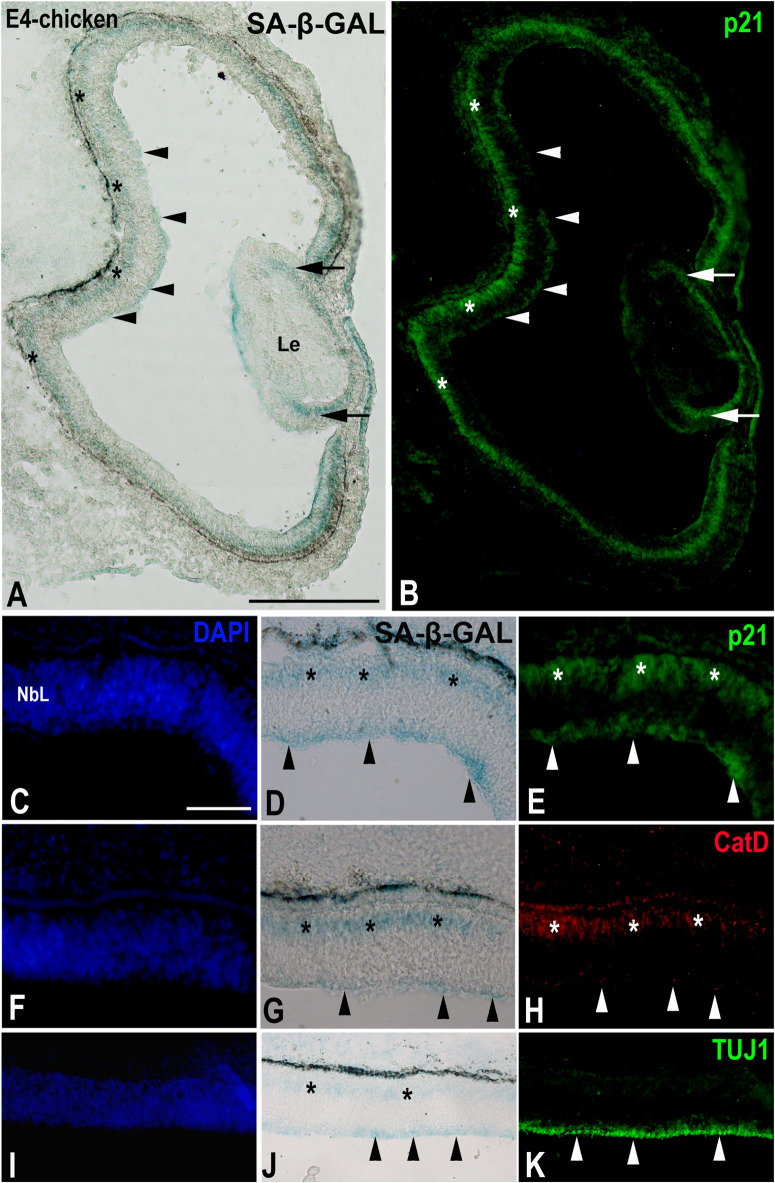
The presence of SA-β-GAL activity in the embryonic day E4 chicken retina. Cryosections of retinas were treated with SA-β-GAL histochemistry and antibodies against p21 **(A–E)**, CatD **(F–H)**, and TUJ1 **(I–K)**. DAPI staining shows that the neural retina consists of a NbL **(C,F,I)**. SA-β-GAL staining is detected in the scleral (asterisks in **A**,**D**,**G**,**J**) and vitreal (arrowheads in **A**,**D**,**G**,**J**) regions of the retina. p21 immunostaining correlates with the SA-β-GAL staining pattern in the undifferentiated retina (arrowheads and asterisks in **B**,**E**) and lens (arrows in **B**). CatD immunoreactivity (arrowheads and asterisks in **H**) is strongly coincident with the SA-β-GAL histochemistry signal (arrowheads and asterisks in **G**). TUJ1 immunoreactivity is intense in the vitreal surface of the NbL (arrowheads in **K**), coinciding with the vitreal SA-β-GAL histochemistry signal detected in the same region (arrowheads in **J**). Le, lens; NbL, neuroblastic layer. Scale bars: 150 μm **(A,B)**, 50 μm **(C–K)**.

A recent study performed in our laboratory (de Mera-Rodríguez et al., 2019) has shown that, in the laminated avian retina, even at embryonic stages, SA-β-GAL labeling is intense in subpopulations of neurons located in the ganglion cell layer (GCL) and in subpopulations of interneurons mainly located in the amacrine cell layer and the horizontal cell layer (Figures 3A,B). Therefore, SA-β-GAL activity is intense in recently differentiated and mature retinal neurons. We also found that SA-β-GAL labeling strongly correlates with p21 immunoreactivity in both the laminated (Figures 3B–D) and the undifferentiated (Figures 4A–E) retina, even in the lens tissue (Figures 4A,B) (de Mera-Rodríguez et al., 2019). SA-β-GAL staining in the non-laminated retina is mainly restricted to the vitreal and scleral surfaces of the neuroblastic layer (NbL) (Figures 4A,D,G,J), strongly correlating with cathepsin D immunoreactivity (Figures 4F–H) (de Mera-Rodríguez et al., 2019), a marker for increased lysosome number or activity (Kurz et al., 2000; Wassélius et al., 2003; Ahuja et al., 2008; Bejarano-Escobar et al., 2011; Lorda-Díez et al., 2019). The SA-β-GAL activity located in the vitreal surface of the retina is detected in TUJ1-positive newborn ganglion cell neuroblasts (Figures 4I–K). Therefore, SA-β-GAL labeling in the developing visual system correlates strongly with the location of the lysosomal mass, but also with other senescence markers, and seems to be linked to neuronal differentiation.

**FIGURE 5:**
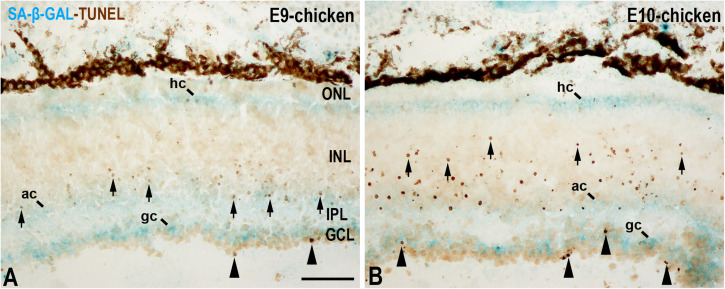
SA-β-GAL activity and cell death in the embryonic day E9 **(A)** and E10 **(B)** chicken retina. Cryosections were doubly stained with SA-β-GAL histochemistry and TUNEL technique. TUNEL-positive nuclei are mainly detected in the GCL (arrowheads) and in the middle region of the INL (arrows). SA-β-GAL activity is observed in the GCL, amacrine cell layer, and horizontal cell layer. ac, amacrine cells; gc, ganglion cells; GCL, ganglion cell layer; hc, horizontal cells; INL, inner nuclear layer; IPL, inner plexiform layer; ONL, outer nuclear layer; pos, photoreceptor outer segments. Scale bar: 50 μm.

But is SA-β-GAL activity also linked to apoptotic cells in the developing retina? In the undifferentiated vertebrate retina, apoptotic cells are either dispersed throughout the NbL or concentrated in areas that surround the optic nerve head (Mayordomo et al., 2003; Francisco-Morcillo et al., 2004; Rodríguez-Gallardo et al., 2005; Valenciano et al., 2009; Bejarano-Escobar et al., 2011, 2013). However, SA-β-GAL activity is mainly concentrated in the vitreal and scleral surfaces of the avian NbL (de Mera-Rodríguez et al., 2019). Later, in the laminated retina, apoptosis follows spatiotemporal patterns that are analogs of the cell differentiation pattern (Figure 5) (Cook et al., 1998; Marín-Teva et al., 1999). In the E9 chicken retina, cell death is mainly concentrated in the GCL and in the amacrine cell layer (Figure 5A) (Cook et al., 1998), and, at E10, TUNEL-positive bodies spread vitreally to the bipolar cell layer (Figure 5B). By these stages, SA-β-GAL staining is detected homogeneously in the GCL, amacrine cell layer, and horizontal cell layer (Figure 5) (de Mera-Rodríguez et al., 2019). There is therefore no correlation between SA-β-GAL activity and the chronotopographical distribution of dying cells in the developing avian retina. In this case, β-GAL activity at pH 6.0 seems to be related to terminal cell differentiation rather than to cellular senescence.

## Conclusion

SA-β-GAL activity increases with aging in neurons of the mammalian brain (for a review, see Walton and Andersen, 2019). In the developing limb, this SA-β-GAL activity correlates well with cellular senescence in the areas of programmed cell death that occur physiologically during the development process. Consequently, a proper balance between senescence and apoptosis is needed for accurate formation of the limbs (Lorda-Díez et al., 2015). However, intense SA-β-GAL is also detected in cells that have intrinsically high lysosomal β-GAL activity, such as macrophages, but also in a wide range of post-mitotic cells, including neurons, even at early stages of embryonic development. Other senescence-associated markers, such as p21, are also activated in parallel with SA-β-GAL activity in recently differentiated retinal neurons (de Mera-Rodríguez et al., 2019). It is likely that there are several common mechanisms involved in both the acquisition of the senescent phenotype and the maintenance of long-term non-dividing cells’ non-proliferating status. Therefore, it is important to discriminate between senescent cells and post-mitotic cells in studies about aging of the CNS because some accepted markers of senescence (SA-β-GAL, p21 expression) are less specific than originally was expected. The identification of new candidate biomarkers of cellular senescence would, in combination with established markers, increase the specificity and efficiency of detecting senescence *in vivo* and *in vitro*. A future requirement will be to have markers for senescence-like phenotypes in long-term non-proliferating cells, such as neurons.

## Author Contributions

GÁ-H, JM-R, YG, GM-P, JR-L, and JF-M wrote and critically reviewed the manuscript. All authors approved the final manuscript.

## Conflict of Interest

The authors declare that the research was conducted in the absence of any commercial or financial relationships that could be construed as a potential conflict of interest.
